# Surmounting the obstacles that impede effective CAR T cell trafficking to solid tumors

**DOI:** 10.1002/JLB.1MR0520-746R

**Published:** 2020-07-03

**Authors:** Emmanuel Donnadieu, Loïc Dupré, Lia Gonçalves Pinho, Vinicius Cotta‐de‐Almeida

**Affiliations:** ^1^ Université de Paris Institut Cochin, INSERM, U1016, CNRS, UMR8104 Paris France; ^2^ INSERM, UMR1043 Centre de Physiopathologie de Toulouse Purpan Toulouse France; ^3^ CNRS, UMR5282 Toulouse France; ^4^ Université Toulouse III Paul‐Sabatier Toulouse France; ^5^ Laboratory on Thymus Research, Oswaldo Cruz Institute Oswaldo Cruz Foundation (Fiocruz) Rio de Janeiro Brazil; ^6^ National Institute of Science and Technology on Neuroimmunomodulation Oswaldo Cruz Institute Rio de Janeiro Brazil

**Keywords:** cancer immunotherapy, T cell trafficking, chimeric antigen receptor engineering, motility, extracellular matrix, tumor microenvironment

## Abstract

Innovative immunotherapies based on immune checkpoint targeting antibodies and engineered T cells are transforming the way we approach cancer treatment. However, although these T cell centered strategies result in marked and durable responses in patients across many different tumor types, they provide therapeutic efficacy only in a proportion of patients. A major challenge of immuno‐oncology is thereby to identify mechanisms responsible for resistance to cancer immunotherapy in order to overcome them via adapted strategies that will ultimately improve intrinsic efficacy and response rates. Here, we focus on the barriers that restrain the trafficking of chimeric antigen receptor (CAR)‐expressing T cells to solid tumors. Upon infusion, CAR T cells need to home into malignant sites, navigate within complex tumor environments, form productive interactions with cancer cells, deliver their cytotoxic activities, and finally persist. We review the accumulating evidence that the microenvironment of solid tumors contains multiple obstacles that hinder CAR T cells in the dynamic steps underlying their trafficking. We focus on how these hurdles may in part account for the failure of CAR T cell clinical trials in human carcinomas. Given the engineered nature of CAR T cells and possibilities to modify the tumor environment, there are ample opportunities to augment CAR T cell ability to efficiently find and combat tumors. We present some of these strategies, which represent a dynamic field of research with high potential for clinical applicability.

## Graphical Abstract

Examines the barriers that restrain the trafficking of chimeric antigen receptor (CAR)‐expressing T cells to solid tumors and potential strategies to overcome these hurdles.

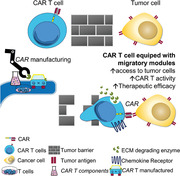

**Abbreviations**
CAR: chimeric antigen receptor
DC: dendritic cell
ECM: extracellular matrix
HER‐2: human epidermal growth factor receptor
PD‐1: programmed cell death‐1
PKA: cAMP‐dependent protein kinase A
RIAD: regulatory subunit I anchoring disruptor
TAM: tumor‐associated macrophages
TCF1: T cell factor‐1
TIL: tumor‐infiltrating lymphocyte
TIM3: T cell immunoglobulin and mucin domain‐containing protein 3
Treg: regulatory T cell
T_RM_: tissue‐resident memory T cell
T_SCM_: T memory stem cell
VEGF: vascular endothelial growth factor
VEGFR‐2: VEGF receptor‐2

## 1 INTRODUCTION

T cells and especially cytotoxic CD8^+^ T lymphocytes (CTLs) have long been recognized to be important in limiting the development of immunogenic tumors.^1^ The presence of CTLs within many tumors is hence a positive prognostic factor.^2^ Conversely, impaired antitumoral immune response is a hallmark of growing tumors.^3^ The concept of T cell‐driven immunosurveillance against cancer has led to the development of immunotherapies based either on the reinvigoration of T cell function in situ, mainly via antibodies targeting immune checkpoint receptors, or on the transfer of genetically modified autologous T cells with enhanced antitumoral activity, mainly chimeric antigen receptor (CAR)‐expressing T cells.^4^ Both strategies have provided an unprecedented level of long‐term antitumor activity in patients with several metastatic cancers. However, the majority of patients with advanced cancers still do not experience sustained clinical benefit from immunotherapy, highlighting the presence of barriers that one needs to identify in order to design strategies that overcome them. Ineffective T cell migration and, in particular, penetration into the tumor mass might represent an important obstacle to T cell based immunotherapies. As a support for this notion, various clinical studies have shown that tumors enriched in T cells are more susceptible to be controlled by programmed cell death‐1 (PD‐1) blockade. In contrast, tumors with so‐called “immune deserts” and immune excluded profiles, in which T cell are present within tumors but not in contact with malignant cells, are refractory to PD‐1 blockade.^5^ Migration might represent an even greater challenge for CAR T cell therapy, because the in vitro expanded T cells that are infused into the blood circulation need to home to the site of tumor development and then migrate toward the tumor mass.

There is currently a wide gap in our knowledge of the homing and migratory properties of CAR T cells, as well as to the location of these therapeutic cells over prolonged periods. The objective of this review is therefore to address key open questions, such as: what are the capacities of infused therapeutic T cells to home to target organs? How does the tumor microenvironment influence the motility behavior of engineered T cells? What are the strategies, which have been implemented to restore a defective CAR T cell migration? How should homing and motility properties of adoptively transferred T cells be monitored in preclinical models? By highlighting these points, we hope to stimulate a research focus at the interface between basic T cell biology and therapeutic development that will ultimately open new opportunities to improve antitumoral T cell based strategies.

## 2 T CELL SUBSETS, LOCATION, AND MOTILITY IN THE CONTEXT OF NATURAL RESPONSES TO CANCER

The natural development of T cell responses against tumors is a highly regulated process that involves multiple steps occurring at different locations. A prerequisite is the expression of antigens, which are specific of the tumor and may result from gene mutations or aberrant gene expression.^6^ Dendritic cells (DCs) present at the tumor site may capture such tumor antigens, for example, in the context of tumor cell apoptosis, before migrating to draining lymph nodes and presenting the antigens to T cells.^7^ In particular, the migratory conventional type 1 DC subset is able to cross‐present antigens to CD8^+^ T cells via MHC‐I molecules.^8^ Alternatively, tumor cells may translocate to lymph nodes draining the primary tumor and deliver antigens directly at this site.^9^ Another possible scenario for T cell priming against tumor antigens is represented by the assembly of tertiary lymphoid organs directly at the site of tumor development.^10^ Owing to the expression of TCRs with enough affinity for the presented antigens, T cells will get activated,^11^ leading to their clonal expansion and to the maturation into effector T cells. In particular, CD8^+^ T cells will acquire cytotoxic function via the expression of effector molecules such as perforin and granzymes. Effector T cells will also acquire the expression of adhesion molecules and chemokine receptors allowing them to migrate to peripheral tissues.^12^ In fact, these effector cells are preferentially attracted to sites of inflammation, such as those associated with tumor growth. However, as compared to infectious settings, T cell trafficking to solid cancers might be inefficient, in part because of a mismatch in homing molecules and receptors, down‐regulation of adhesion molecules, and aberrant vasculature.^13^ A side effect to this might be the relocation of tumor‐specific T cells to other sites, as effector T cells can reach different inflamed tissues, regardless of the site of initial antigenic stimulation. The ability for tumor‐specific effector T cells to gain access to sites of tumor metastasis appears to be crucial to the control of cancer spreading^14^ and possibly to maintain new seeds of cancer cells in a state of dormancy.^15^, ^16^ It is however unclear whether effector T cells may act inside the vasculature to control circulating cancer cells or at the site of distant seeding. The ability of immune checkpoint therapy to control the metastatic process has been documented in several clinical trials.^17^, ^18^ However, whether this applies to CAR T cell therapy remains to be determined. In the case of an efficient effector T cell response, the antigenic trigger is cleared and a fraction of effector T cells develop into peripheral memory T cells that can reside in the target tissue for prolonged periods, as a means to provide protection against a secondary occurrence of the antigenic trigger. However, in the context of cancer, the tumor cells as well as their microenvironment offer resistance to the effector function of T cells and resolution is rarely achieved. This is associated with the development of T cells within tumors that exhibit exhaustion features.^19^, ^20^ Exhaustion corresponds to various T cell differentiation states usually associated with chronic antigenic stimulation and with reduced function as compared to effector cells. Whether exhausted T cells may also harbor altered motility and homing properties remains to be investigated. In this context, the differentiation status of therapeutic T cells generated by in vitro stimulation and specific culture conditions is expected to highly condition their fate in vivo, including their effector functions toward the tumor and most probably their trafficking properties.

Defining and generating the population of T cells with the highest potential to control tumor growth is of pivotal importance for the design of T cell based therapies. The rare subset of T memory stem cells (T_SCM_) is of particular relevance, because of its self‐renewal capacity and ability to differentiate into effector cells.^21^, ^22^ T_SCM_ harbor a unique combination of naïve T cell markers and memory markers such as CD58, CD95, IL‐2Rβ, and CXCR3. A cell tracking study, performed in patients having received genetically modified T cells in the context of gene therapy trials for ADA‐SCID, has highlighted that T_SCM_ can persist for up to 12 yr post‐infusion and that such cells maintain they precursor potential.^23^ Single cell analysis of T cell populations infiltrating different human cancers has recently uncovered the presence of T cells endowed with some level of stemness. In the context of nonsmall cell lung cancer, stem‐like CD8^+^ T cells, harboring a partial exhausted phenotype together with effector potential and self‐renewal capacity, have been identified.^24^ However, these cells lack the canonical T_SCM_ phenotype of circulating T cells. In favor of the notion that these cells play a protective activity, they are lost with disease progression. Analysis of a cohort of patients with kidney cancer showed that T cell infiltrates are composed of T cell factor‐1 (TCF1)^+^ stem‐like and T cell immunoglobulin and mucin domain‐containing protein 3 (TIM3)^+^ terminally differentiated T cell subsets.^25^ These subsets harbor proliferative potential and cell killing potential, respectively. Furthermore, TCF1^+^ stem‐like T cells give rise to TIM3^+^ terminally differentiated T cell upon stimulation. Interestingly, TCF1^+^ CD8 T cells are located in regions enriched with MHC‐II^+^ cells across various human cancers, suggesting that stem‐like CD8^+^ T cells require a lymphoid‐like environment and are key to sustain the terminally differentiated T cell population mediating the antitumor immune response. Another T cell subset of interest for the prospects of T cell therapy is represented by CD8^+^ tissue‐resident memory T (T_RM_) cells, which have been shown to constitute a prevalent effector population in the microenvironment of primary triple‐negative breast cancer.^26^ Indeed, a T_RM_ gene expression signature is associated with improved relapse‐free and overall survival after standard chemotherapy, implying a beneficial antitumor function for this subset. In this context, such cells display reduced expression of tissue egress genes such as *S1PR1* and *KLF2*. Residency within the tumor might therefore be considered a desired property for the design of T cell based therapies.

## 3 T CELL HOMING AND MIGRATION IN THE CONTEXT OF CAR T CELL THERAPY

### 3.1 Principles of adoptive T cell therapy

Adoptive T cell therapy is based on the isolation, in vitro expansion, and reinfusion of autologous CD8^+^ T cells endowed with cytotoxic activity against cancer cells. The therapeutic cell population consists of either tumor‐infiltrating lymphocytes (TIL) or genetically modified T cells expressing a CAR specific for a expressing a CAR specific for a tumor antigen. Although TIL infusion has been the first approach to demonstrate the efficacy of T cell based immunotherapy,^27^ most current approaches focus on CAR T cell development because of the less restricted access to primary cells and the modulatory potential of the genetic engineering.^28^

CARs have a modular structure with four domains: an antigen‐binding region, a hinge, a transmembrane domain, and an intracellular signaling domain. Excellent reviews have been published on the function of each of these elements.^29^, ^30^, ^31^ Here, we place greater emphasis on the signaling domain as it can impact T cell activities including motility. First‐generation CARs were composed, in their signaling domains, of CD3ζ‐ derived immunoreceptor tyrosine‐based activation motifs. T cells engineered with these CARs were able to bind to and kill their targets. However, these cells failed to persist in cancer patients. A major improvement came with the addition of costimulatory domains derived from CD28 or 4‐1BB (CD137) in so‐called second‐generations CARs. Recent studies suggest that CD28‐based CARs have greater initial antitumor activity, whereas 4‐1BB signaling enhances CAR‐T cell persistence and reduces exhaustion.

CAR T cells recognizing CD19 with 4‐1BB domain (marketed as Kymriah) have been approved for the treatment of patients up to 25 yr of age with relapsed/refractory B cell acute lymphoblastic leukemia. On the other hand, CD19 CAR T cells with CD28 domain (marketed as Yescarta) have been approved for adult relapsed or refractory large B cell lymphoma. In the past few years, clinical trials using these CAR T cells on malignant/leukemic B cells have shown high rates of response (70–90%) that are unprecedented, especially in relapsed and refractory acute B cell leukemia.^32^ Nonetheless, the field of CAR T cells is currently facing two major challenges. First, although CAR T cells can be extremely effective in killing malignant cells, they can also cause deleterious side effects, including off‐tumor toxicity and cytokine release syndrome.^33^ Second, the CAR T cell strategy has not yet produced favorable clinical responses in targeting solid malignancies.^34^ Up to now, most clinical trials on CAR T cells in solid tumors indicate no or scant objective responses (see Fuca et al.^35^ for a comprehensive presentation of clinical trials on solid tumors). This is, for example, the case of sarcoma patients infused with human epidermal growth factor receptor (HER)‐2 CAR T cells,^36^ or pancreatic carcinoma patients infused with mesothelin CAR‐T cells.^37^ Most worrisomely, severe toxicities caused by on‐target but off‐tumor antigen recognition by CAR T cells have been reported. In one study, the infusion of CAR T cells targeting HER‐2 caused fatal acute respiratory distress syndrome due to recognition of lung epithelia cells expressing low levels of HER‐2.^38^

### 3.2 Homing, migration, and persistence of therapeutic T cells in target tissues

**FIGURE 1:**
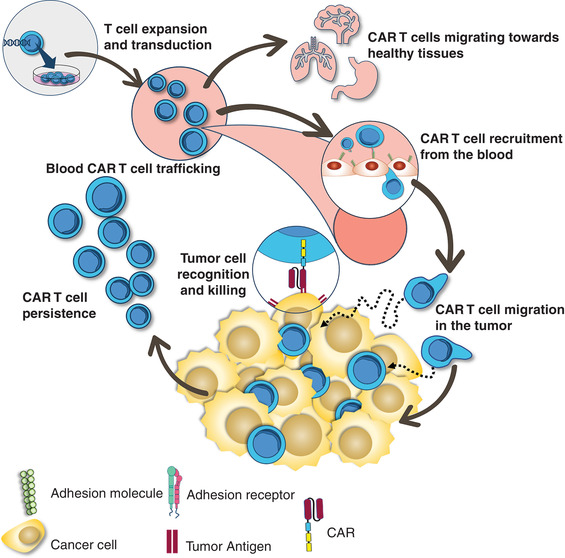
**Homing, migration and persistence of chimeric antigen receptor (CAR) T cells in tumors**. CAR T cell therapy comprises T cell isolation followed by a CAR transduction and in vitro expansion of the CAR T cells. After the T lymphocytes are reinfused, they need to be recruited from the blood to the tumor site. There, the engineered receptors are able to interact with specific antigens present on tumor cells and, ultimately allowing the cytotoxic killing. Additionally, persistence of CAR T cells either in the tumor site or in the peripheral blood is critical for robust clinical responses

As stated earlier, CAR T cells have shown remarkable success in B cell malignancies. Many of these semiliquid hematologic malignancies reside in the bone marrow, a niche easily accessible for intravascular T cells. In solid tumors, the situation is strikingly different and CAR T cells face additional barriers that lymphocytes must break in order to control tumor growth (Fig. 1). First, CAR T cells need to home into the tumor during the extravasation phase by crossing tumor blood vessels. A study performed in a metastatic breast cancer patient with HER‐2‐specific T cells demonstrated the incapacity of infused lymphocytes to home into solid tumor masses. This is in sharp contrast with the accumulation of tumor‐specific T cells into the cancer patient's bone marrow.^39^ Similar results obtained in preclinical mouse tumor models led to the conclusion that T cell homing into solid tumors is a limited process.^13^ Key factors for T cell entry into tumor masses are chemokine receptors and adhesion molecules. We know that active trafficking of T cells into tumors partially depends upon the compatibility between chemokines found in tumor and chemokine receptors expressed on T cells. Once T cells enter the tumor, they must disseminate and physically contact their targets (Fig. 1). In carcinomas, which represent 90% of solid tumors, tumor cells are organized in islets surrounded by specialized microenvironment, referred to as the stroma. As tumor blood vessels are localized in the stroma, newly entered T cells must migrate from their entry points to their targets. This interstitial migration is influenced by many external elements such as soluble factors as well as by cellular and structural determinants.

Few studies have documented the navigation of T cells within the tumor stroma. By using a model of fresh human tumor slices combined with dynamic imaging microscopy, we have monitored the motility of resident T cells in human solid tumors. Overall, we found that T cells migrate poorly in the stroma of human lung tumors (2.5 μm/min of mean velocity from 9 patients) highlighting the presence of obstacles that impede T cells from reaching cancer cells.^40^ This notion is reinforced as, in few patients whose tumor islets are enriched in T cells, lymphocytes move fast in the stroma.

After their navigations within the stroma, T cells need to form productive conjugates with their targets (Fig. 1). Imaging experiments have provided insights in the way T cells engage malignant cells. At the molecular levels, we know that together with the binding of the TCR with peptide‐MHC complexes, adhesion molecules are important.^41^ Among these, two integrins, namely LFA‐1 and αEβ7, play a critical role in T cell/tumor cell interaction. LFA‐1 binds to ICAM‐1 (CD54) expressed by antigen‐presenting cells as well as by some tumor cells.^42^ When T cells are stimulated through their TCRs or chemokine receptors, the affinity and clustering of LFA‐1 increases in an inside‐out signaling process that promotes the binding of this integrin to ICAM‐1. Whether similar molecules and mechanisms control the antitumoral action of CAR T cells remains to be established.

In vitro and in vivo experiments have also documented the kinetic interactions between T cells, including CAR T cells and tumors cells. In vitro experiments performed with murine CAR T cells indicate that lymphocytes sequentially contact and kill several tumor cells, with faster formation of a synapse and more rapid detachment of tumor cells compared with TCR‐mediated cytotoxicity.^43^, ^44^ In vivo experiments support the notion of fast killing dynamics for CAR T cells, which would contribute to their therapeutic efficacy. Indeed, CD19 CAR T cells were observed to engage, kill, and detach from malignant B cells in ∼25 min.^45^ This rapid detachment that would favor serial killing contrasts with previous observations of conventional CD8^+^ T cells forming long‐lasting interactions with tumor cells.

Finally, to successfully control the tumor, CAR T cells need to persist for a long time in cancer patients (Fig. 1). In patients with hematologic malignancies, it has been shown that the persistence of CAR T cells in peripheral blood is an essential factor for durable clinical response. It is likely that the same holds true for solid tumors, although no evidence is available for the moment. The determinants that control T cell persistence are actively searched and linked to epigenetic factors controlling the lymphocyte differentiation process.^46^ In particular, the process of T cell exhaustion associated with chronic antigenic activation and specific epigenetic marks may account for the restricted efficacy of CAR T cells. As recently reported, overexpression of the transcription factor JUN in CAR T cells is a promising strategy to counteract their exhaustion and improve their therapeutic efficacy.^47^

Optimally, CAR T cell therapy should combine the activity of cells with immediate cytolytic effector function to kill the bulk of fast‐growing tumors and the persistence of tumor‐specific cells with self‐renewal capabilities to provide a prolonged supply of cytolytic effector progeny to ensure long‐term control over tumor cells. Current in vitro methods employed to expand cells to sufficient numbers and still maintain a minimally differentiated phenotype are hindered by the biologic coupling of clonal expansion and effector differentiation.^48^ Therefore, a better understanding of the physiologic mechanism that couples cell expansion and differentiation of CD8^+^ T cells may improve the efficacy of T cell based immunotherapy.

## 4 BARRIERS TO T CELL MOTILITY IN THE CONTEXT OF IMMUNOTHERAPY

### 4.1 Defects in T cell entry

**FIGURE 2:**
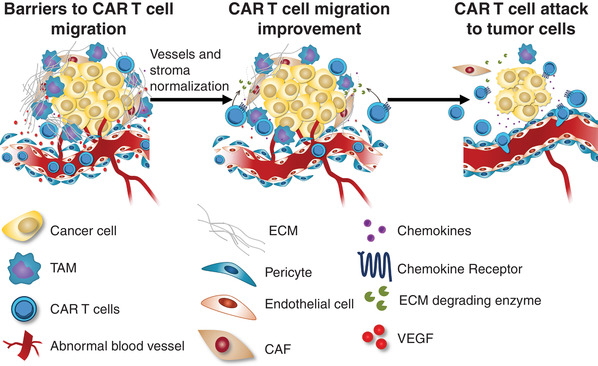
**Barriers and improvements to chimeric antigen receptor (CAR) T cell migration**. CAR T cells should overcome the barriers to cell migration at tumor sites in order to perform their cytolytic effector function. Obstacles to T cell trafficking toward tumors might occur due to abnormal tumor blood vessels with pericyte detachment, dysregulation of chemokine‐chemokine receptor interaction, deposition of extracellular matrix (ECM) proteins by cancer‐associated fibroblasts (CAF), as well as encounter with tumor‐associated macrophages (TAM). Therefore, targeting of tumor micro‐environmental components represents an important approach for CAR T cell therapy improvement. The combination of chemokine‐chemokine receptor signaling with co‐expression of ECM degrading enzymes could be determinant for T cell function, as well as enhancing the therapeutic efficacy of CAR T cells

The endothelium is a key checkpoint to control leukocyte recruitment during an inflammatory response. Particularly, T cells will have to cross the vasculature in order to access the target tissue. A number of factors are involved in this step, including chemokines and adhesion molecules responsible for the tethering, rolling, adhesions and transmigration phases through the activated endothelium. Aberrant vasculature operates as a first hindrance for T cell trafficking into the tumor sites (Fig. 2, left panel). Tumor endothelium is regarded as structurally immature and disorganized, with a critical role for the higher activity of pro‐angiogenic factors, which is associated to abnormal blood flow and permeability, ultimately leading to increased tissue pressure, collapse of the blood vessels, and dysregulated oxygen supply.^49^ The pericytes, important cells in vessel maintenance and development, show structural changes in cancer. Tumor pericytes appear to be detached from the vessel structures, thereby modifying angiogenic signaling.^50^ Additionally, the increased presence of angiogenic growth factors induces endothelial cell anergy, a state of low inflammatory response in the tumor site as a result of decreased expression of chemokines as well as molecules important for the adhesive activity of immune cells on the tumor endothelium.^51^, ^52^ In addition, T cells often do not express the chemokine receptors for the chemokines produced by tumors. For CAR T cells, a recent study using a positron emission tomography probe to assess lymphocyte trafficking did find the presence of intravenously injected engineered cells in mouse bearing tumors positive for the disialoganglioside GD2.^53^ However, the accumulation of CAR T cells into tumors was relatively slow, peaking 2 wk after the initial transfer. This access problem is even magnified when dealing with brain tumors, forcing investigators to circumvent the blood‐brain barrier by intracranial injection.

### 4.2 Defect in T cell migration within tumors

In many tumors, although lymphocytes have successfully crossed tumor blood vessels, T cells are not in contact with malignant cells but instead enriched in the surrounding stroma.^54^ This so‐called “excluded immune infiltrate” phenotype is associated with a lack of responsiveness to PD‐1 blockade, stressing the importance of stromal obstacles that need to be identified in order to overcome them. The reasons for this T cell sequestration in the tumor stroma are manifold, as pointed out below.

#### 4.2.1 Extracellular matrix (ECM) barrier

Increase in tumor stiffness is a well‐established feature of growing tumors. This is due to an increased deposition and aberrant architecture of tumor ECM proteins as well as an important presence of cancer‐associated fibroblasts.^55^ This desmoplastic state has been shown to contribute to immune suppression through various mechanisms. By using dynamic imaging microscopy combined with the experimental system of tumor slices made from human tumors, we have demonstrated a detrimental impact of ECM on T cell migration and on their ability to reach tumor cells (Fig. 2, left panel). More precisely, a thick network of collagen fibers surrounding some tumor islets may constitute a physical obstacle for T cell lymphocytes to infiltrate tumor cell areas.^56^

#### 4.2.2 Myeloid cells

Myeloid cells are very abundant cells within tumors. These cells engage T cells in long‐lasting, unproductive interactions, potentially acting as a functional barrier to T cell antitumoral activities (Fig. 2, left panel).^57^ In a spontaneous murine model of breast cancer, myeloid cells sharing some analogies with both DCs and tumor‐associated macrophages (TAM) have been shown forming long‐lasting contacts with T cells.^58^ Our recent data obtained in human and murine tumors confirm similar stable conjugates formed between TAM and CD8^+^ T cells in the stroma.^40^ This cell‐cell interaction might sequester CTLs in the stroma, excluding them from the vicinity of cancer cells. Important questions remain concerning the mechanisms by which TAM prevent CTLs from reaching tumor cells. In addition, it is not known for the moment whether CAR T cells also engage myeloid cells in long‐lasting interactions within tumors.

#### 4.2.3 Hypoxia

Hypoxia, a feature of growing tumors, has been shown to dampen T cell functions.^59^ Low oxygen tension can lead to metabolic stress, which in turn suppresses T cell responses including lymphocyte abilities to migrate. Two‐photon imaging studies have demonstrated the dependence of T cells to oxygen for their migration. This applies to lymphoid tissues and also to tumors. The monitoring of CTL behavior in mouse melanoma tumors using intravital two‐photon microscopy has indicated that CD8^+^ T cells accumulate and migrate preferentially in perivascular areas.^60^ Conversely, T cells localized far from blood vessels exhibited a slow mobility. Of interest, reducing hypoxia in mouse tumors has been shown to restore T cell infiltration and increase the efficacy of anti‐PD‐1 therapy.^61^ No data is yet available concerning an impact of hypoxia on CAR T cell antitumoral activities. Nevertheless, the CAR costimulatory domains (CD28 vs. 4‐1BB) may impact differently on T cell mitochondria and oxygen consumption,^62^ but how this affects intratumoral motility is unknown. Apart from hypoxic conditions, altered levels of metabolic factors (e.g., reduced glucose, increased lactate) are likely to affect the T cell motility within tumors as well.

#### 4.2.4 Chemokines

Chemokines, a group of cytokines with chemotactic activity, have been regarded as critical players in several aspects of the tumor progression, such as angiogenesis, metastasis, and leukocyte migration into tumor sites. Several studies have mapped their role in regulating recruitment and the subsequent pro‐ or antitumoral activity of distinct leukocyte subpopulations, such as TAM and neutrophils, DCs, regulatory T (Treg) cells, as well as effector T cells.^63^ In this context, together with a limited T cell access due to the stromal barrier, a lack of pro‐migratory factors has also been suggested to explain the paucity of T cells found in many tumors. We have now a better understanding of the nature of the chemokines important for T cell recruitment into tumors. In multiple human tumors, CCL5 and CXCL9 are well correlated with CD8^+^ T cells.^64^ Whereas CCL5 is constitutively expressed by tumor cells, CXCL9 is expressed by DCs and inflammatory macrophages in response to IFN‐γ. Moreover, several mechanisms have been identified in the down‐regulation of CCL5 expression by malignant cells, including the WNT/β‐catenin pathway.^65^ Of note, some chemokines can also have a detrimental impact on T cell migration and ability to contact tumor cells. For example, in a pancreatic mouse tumor model, production of CXCL12 by stromal fibroblasts impedes T cells from infiltrating tumor islets by a mechanism, which remains to be clarified.^66^

### 4.3 Defect in synapse formation between T cell and malignant cells

Once T cells have passed the stromal obstacles described earlier, they need to form productive contacts with malignant cells. Recent data indicate that the density of the target antigen plays a critical role in CAR T cell efficacy. In B leukemia patients treated with CD19 CAR T cells, despite initial high complete response rates, relapses occur in a large fraction of patients.^67^ The loss of the target antigen at the surface of malignant B cells is recognized as being a major cause of relapse. Logically, CAR T cells would not be able to establish productive immunologic synapses with malignant cells that have lost their target antigens. More surprisingly is the observation that tumor cells in which the target antigen has decreased but not disappeared are not efficiently eliminated with CAR T cells. For example, a significant percentage of B leukemia patients infused with CD22 CAR T cells relapses from the treatment despite a residual expression of CD22 by cancer cells.^68^

Similar conclusions were drawn from experiments conducted in preclinical mouse tumor models. Tumor cells with intermediate expression of the target antigen were not efficiently eliminated by CAR T cells.^69^, ^70^ These observations led to the conclusion that CARs, although of high affinity for their targets, are not very sensitive and require a high amount of antigen at the cancer cell surface. This is in sharp contrast with the functioning of TCRs, which are highly sensitive receptors able to recognize a very limited numbers of peptide‐MHC complexes.^71^ The molecular mechanisms underlying the low CAR sensitivity is actively searched with attention paid to the signaling elements of the chimeric receptor. Hence, a recent study has demonstrated that for low antigen densities, CARs with CD28 signaling domains were much more potent than CARs with 4‐1BB signaling domains.^72^ Moreover, strategies aiming at increasing the surface expression of the antigen targeted by the CAR have been recently implemented to prevent resistance to CAR T cell therapy.^73^ Altogether, the notion that emerges from these findings is that progressing tumors have accumulated multiple mechanisms impeding T cells from migrating and contacting tumor cells.

### 4.4 CAR T cell aberrant migration

Apart from a defective migration into and within the tumor, effector T cells can also exhibit aberrant distribution, which can lead to severe toxicities. Cytokine‐release syndrome and CAR T cell‐related encephalopathy syndrome are the two most‐common life‐threatening toxicities observed after CAR T cell therapy, although rare cases of anaphylaxis have also been reported.^74^ The pathophysiologic mechanism underlying these toxicities remains to be determined. Nevertheless, accumulating evidence suggests that neurotoxicities are associated with a disruption of the blood‐brain barrier and the infiltration of CAR T cells into the CNS.^75^, ^76^, ^77^, ^78^ These events lead to elevated levels of inflammatory cytokines in the cerebrospinal fluid responsible to an inflammation of the CNS. But CAR T cells can also be found in other organs, as suggested by the monitoring of CD19 CAR T cells in mouse developing lymphomas, which revealed evidence for trapping of transferred cells into the lungs.^45^ The underlying mechanisms of such cellular distributions are complex, with the expression of variable amount of CAR‐targeted antigen recognized by engineered T cells. Thus, any approaches implemented to correct a defective intratumoral migration should be carefully weighed to prevent excessive CAR T cell migration in off‐site tumor places.

## 5 REWIRING T CELL MOTILITY AS AN OPPORTUNITY TO IMPROVE IMMUNOTHERAPY

Along its journey toward a specific tumor target, a T cell will face complex interactions with distinct tissue barriers, such as blood vessels, the tumor microenvironment, as well as the tumor tissue itself. Thus, directly targeting molecular components involved in T cell adhesive and motility activities, might overcome tissue obstacles for efficient trafficking and lead to improvement of CAR T cell therapy (Fig. 2, middle panel).

### 5.1 Targeting tumor vasculature to improve T cell migration

An abnormal expression of angiogenic growth factors is a hallmark of growing tumors. Therefore, anti‐angiogenic strategies aimed at normalizing the structure of tumor vasculature, and a likely subsequent reversal to an increased expression of endothelial adhesion molecules, might decisively enhance T cell recruitment and response to tumor tissues.^79^ In fact, in a murine melanoma model, disruption of vascular endothelial growth factor/VEGF receptor‐2 (VEGF/VEGFR‐2) signaling by treatment with anti‐VEGF showed a synergistic efficiency with an immunotherapy employing adoptively transferred T cells carrying a transgenic TCR directed to a melanoma antigen, resulting in enhanced tumor tissue infiltration.^80^ Moreover, in another murine cancer model, combined neutralization of VEGF/angiopoietin‐2 led to improved tumor control, as a result of both better response with anti‐PD‐1 blockade and increased accumulation of perivascular CD8^+^ T cells.^81^ As targeting of VEGF also disrupts its immunosupressive effects on the tumor microenvironment, such a strategy deserves further investigation as a countermeasure to the tumor microenvironment devoid of infiltrating T cells.^82^ Additionally, this approach can also be part of a thoughtful multistrategic therapy for combating cancer. The combination of angiogenic blockade and immunotherapy along with radiotherapy, which also leads to overexpression of adhesion molecules in the target tumor vessels, might represent an effective pathway to enable T cells to access the tumor site.^83^

### 5.2 Targeting cells and extracellular components in tumor microenvironment to improve T cell migration

The tumor microenvironment seems to be a dysregulated battlefield where recruited leukocytes contribute distinctly for antitumoral immunity or for a pro‐tumorigenic microenvironment via modulation of the specific immune response. In fact, an immunosuppressive outcome follows the presence of metabolic alterations and the secretion of immunoregulatory cytokines. Besides, suppressive activities are also exerted by distinct subpopulations infiltrating the tumor site, such as Treg cells and myeloid cells.^84^ All these components play a role in limiting the activity of CAR T cells and their subsequent capability to gain access to the tumor tissue. Therefore, controlling the activity of immunosuppressive components associated to the tumor site seems a relevant strategy aimed at improving CAR T cell therapy. In this context, Treg depletion has been regarded as an important mechanism of anti‐CTLA‐4 immunotherapy.^85^ Interestingly, an experimental study showed a significant increase in CD8^+^ T cell infiltration and tumor rejection following extensive Treg depletion, a finding that might be associated to tumor vasculature normalization, which express increased levels of VCAM‐1 and ICAM‐1 on endothelial cells.^86^ Moreover, as recent reports have pointed, other particular cell subsets act as critical players in avoiding intratumoral T cell infiltration. These negative regulators probably affect CAR T cells too, although further studies on specific cell depletion to improve CAR T cell trafficking are warranted. As stated previously, macrophages have a detrimental impact on T cell ability to reach cancer cells, supporting the use of strategies that either deplete or reprogram macrophages. Hence, in several mouse tumor models the depletion of macrophages allowed CD8^+^ T cells to infiltrate and mediate tumor regression when combined with anti‐PD‐1 blockade.^40^, ^87^ This aspect takes an increasing importance, as macrophages have also been associated with some forms of toxicity (e.g., cytokine release syndrome) after CAR T cell infusion.^88^ However, one must keep in mind that macrophages with a proinflammatory phenotype can also exert antitumoral activities either alone or in cooperation with T cells, stressing the risk of a depletion strategy.^89^ Approaches to target cancer‐associated fibroblasts, which are responsible for the excessive production of the ECM limiting intratumoral T cell migration, have been also implemented. In a lung cancer model, a CAR T cell was engineered to successfully deplete cancer‐associated fibroblasts, which comprise a central cellular subset of the tumor microenvironment.^90^ As expected, such a strategy inhibited tumor growth even more significantly by co‐targeting cancer‐associated fibroblasts and the specific tumor antigen. Although the mechanistic basis for this enhanced antitumor activity was not provided, the effective T cell infiltration might be related to normalization in tumor vascularization, mitigation of an immunosuppressive milieu, as well as modulation of the ECM content. Other strategies aim at directly targeting the ECM, because CAR T cells have a limited capability to degrade it and, therefore, might harbor an impaired trafficking toward the tumor tissue (Fig. 2, left panel). In fact, in vitro expansion of CAR T cells leads to down‐regulation of *HPSE* gene and a consequent loss of heparanase, an enzyme that T cells secrete to degrade heparan sulfate proteoglycans in ECM.^91^ By engineering CAR T cells to co‐express heparanase, these cells showed both an improvement in ECM degradation and an increase of in vitro migration activity, as ascertained by a Matrigel‐based cell invasion assay. Moreover, enhanced tumor infiltration and decreased tumor growth correlated with an improved mouse survival. The ECM as a pivotal component of the CAR T cell‐resistant tumor stroma could also be evidenced from the striking observation that CAR T cells specific for chondroitin sulfate proteoglycan 4 (a melanoma surface proteoglycan) demonstrated a relevant antimelanoma activity, whereas co‐targeting strategy of heparan sulfate proteoglycans and CD19 did not seem to be efficient to control the stroma‐poor B cell lymphoma.^91^

The association of classical chemotherapy or radiotherapy strategies to the CAR T cell therapy has been postulated to play a role in targeting the tumor microenvironment, with a potential impact on CAR T cell trafficking. Previously, the effect of ablative radiotherapy upon primary tumor and metastasis was reported to be strongly dependent on CD8^+^ T cell response.^92^ Radiotherapy seems to induce an inflammatory milieu, which might favor increased vascular adhesion and chemotaxis‐driven migration.^93^, ^94^ In addition, irradiation of cancer cell lines led to increased expression of tumor‐associated antigens.^95^, ^96^ Therefore, an efficient CAR T cell migratory and infiltration capabilities might benefit from a combination to a classical radiotherapy to treat solid tumors.

### 5.3 Targeting chemotactic response to improve T cell migration

Targeting of chemokine‐chemokine receptor signaling has been tested in several preclinical and clinical studies. Particularly, T cell trafficking into tumor sites following endothelial transmigration is also governed by a proper response to the chemokine milieu in these sites. In this sense, as effector memory T cells bear high densities of CXCR3 and CCR5 chemokine receptors, it is expected that they are able to infiltrate and target tumors producing chemokines that signal through these receptors. In fact, CCR5 was the first chemokine receptor demonstrated to be involved in the infiltration of cytotoxic T cells to tumor site.^97^ Such a finding could further evolve as a genetic approach to tune the migratory activity of T cells, in order to redirect them toward a given chemokine secreted by tumor cells.^98^ However, poor antitumoral cytolytic T cell function follows a chemokine/chemokine receptor mismatch, as tumors may produce low levels of chemokines or effector T cells may lack the receptors for chemokines specifically expressed in the target tumor. Therefore, a proposed genetic approach was to use CAR T cells expressing chemokine receptors that properly match the chemokines produced by the target tumor (Fig. 2, middle panel). In fact, CAR T cells, which lack the expression of CCR2, when engineered to co‐express this chemokine receptor, migrate and respond promptly against tumors that express higher levels of the chemokine CCL2, a CCR2 ligand.^99^, ^100^ Likewise, co‐expression of IL‐8 chemokine receptor, CXCR2, in CAR T cells resulted in improved in vitro migration and enhanced in vivo antitumor activity and responsiveness to high IL‐8‐producing tumor cell lines.^101^ In an experimental model of Hodgkin lymphoma, a noteworthy strategy took into account that the lymphoma cells predominantly produce CCL17 and CCL22 and recruit CCR4^+^ Th2 and Treg cells, resulting in an immunosuppressive tumor microenvironment. As CCR4^−^ effector CD8 T cells are rarely present at the tumor site, the strategy of generating CAR T cells that co‐express CCR4 resulted in higher in vivo migratory and antilymphoma activities.^102^ It is important to point out that such an approach might also lead to some drawbacks, such as off‐target toxicity, as the chemokines are not restricted to tumor sites, as well as inefficient response, as chronic stimulation through the chemokine receptor might also occur. Alternatively, recent reports demonstrated enhanced antitumoral response by associating a CAR T cell immunotherapy along with specific approaches to increase chemokine expression within tumors. An approach employing CAR T cells along with an oncolytic vaccinia virus engineered to produce CXCL11, a CXCR3 ligand, showed higher efficiency in recruiting CAR T cells and control tumor progression in comparison to the viral therapy alone. Moreover, the authors also demonstrated comparatively that an approach by engineering those CAR T cells to produce CXCL11 was not able to increase T cell migration into tumoral site, despite the increased local chemokine production.^103^

Additionally, immunosuppressive factors, such as PGE_2_ and adenosine, are reported to be enriched in the tumor microenvironment of solid tumors and exert potent inhibition of T cell functions, including migration and adhesion. As this inhibition is dependent on cAMP‐dependent protein kinase A (PKA) activation and its recruitment to the immune synapse by anchoring to the membrane protein ezrin, an interesting approach by targeting intracellular determinants of such an effect was envisaged to improve CAR T cell activity.^104^ Blockage of PKA‐ezrin interaction, by employing the “regulatory subunit I anchoring disruptor” (RIAD), restricted the cAMP/PKA‐mediated immunosuppression and the engineered CAR‐RIAD T cells carry increased antitumor activity in vivo, when compared to CAR T cells. Noteworthy, CAR‐RIAD T cells also showed higher baseline expression of CXCR3 and the adhesion molecule CD49d, a finding that might be, respectively, correlated with their increased CXCL10‐driven chemotaxis and adhesion to fibronectin and VCAM‐1.^104^ This report is pivotal in highlighting intracellular determinants of T cell function as potential targets to engineer enhanced efficacy for CAR T cell therapy.

Besides these modulatory strategies on chemotaxis, it is important to point out the regional delivery of CAR T cells as a potential approach to surmount the obstacles for T cell migration toward the target tumor.^105^, ^106^ By increasing the efficacy of CAR T cells to target solid tumors, such an approach might also limit their off‐tumor toxicity. In fact, both experimental and clinical studies indicate effective action of locally delivered CAR T cells against brain tumors.^107^, ^108^, ^109^, ^110^

## 6 IMPORTANCE OF PRECLINICAL MODELS IN ASSESSING CAR T CELL DYNAMICS

One of the key challenges in the field of cancer immunotherapy, including CAR T cells, is to develop relevant models to test and predict the efficacy and safety of cell products.^111^ As stated earlier in our review, migration largely contributes to the efficacy of CAR T cells but can also lead to toxicity, underlining the need of relevant models to track engineered lymphocytes. Historically, in vitro approaches and mouse tumor models have been extensively used to study CAR T cell migration.

Cell culture methods typically rely on cancer cells or immortalized cells grown within artificial environments admixed with CAR T cells. Although in vitro cell culture models do not integrate the complexity of the tumor environment, they possess a number of advantages. In particular, they are easily amenable to imaging microscopy and, in this context, several reports described the dynamics of CAR T cells during their contact with tumor cell lines.^43^, ^44^, ^112^ It is expected that such models combined with high‐resolution microscopy will particularly be useful to provide insights on the structure of the immune synapse.

Efficacy of CAR T cells is usually tested in mice harboring tumors. The model of choice is the transfer of human CAR T cells in a highly immune‐compromised NOG/NSG mouse (NOD/SCID/ IL‐2Rγ^−/−^) previously transplanted with a human cancer cell line. These models have the benefit of verifying the potency and antitumor activity of engineered T cells by monitoring the size of the tumor during the treatment. Regarding CAR T cell migration, such models have been used to monitor lymphocyte bio‐distribution noninvasively using clinical imaging modalities (e.g., with positron emission tomography reporter gene).^113^ A key limitation of these xenografted models is a lack of immune cells and components that presumably take an important part in controlling the trafficking of CAR T cells. Recently, there has been a marked effort to develop more relevant mouse models to test immunotherapy products. In this regard, humanized mice harboring tumors have been successfully used to evaluate CAR T cell toxicity.^114^

In addition to xenografted models, studies in immune‐competent mice with murine CAR T cells have been conducted. Combined with intravital two‐photon microscopy techniques, they have unraveled the behavior of CD19 CAR T cells in time and space.^45^, ^115^ Nevertheless, although these models provide very valuable information, they do not always recapitulate human biology. For example, the murine immune system, including its composition and phenotype, differs from that in humans. This is important when one deals with toxicities associated with CAR T cells, which have been largely missed in immunocompetent mouse models.^116^

Beyond murine models, embryonic zebrafish xenografts have recently been proposed as an alternative for preclinical evaluation of CAR T cells.^117^ Zebrafish embryos are permissive to human cell transfer including tumor cells and T cells. Because of their size and transparency, they appear particularly suited for tracking the motility and killing activities of CAR T cells with high resolution and high throughput.

Additionally, 3D models, including organoids, are increasingly popular these days. They usually consist of purifying cancer cells from a fresh human biopsy, followed by an aggregation step. Their use in testing immunotherapy reagents, including CAR T cells, is fast‐growing.^118^, ^119^, ^120^ They are patient specific, easily cultured several weeks and amenable to dynamic imaging microscopy. However, they usually lack the structure observed in human carcinomas with compact tumor islets surrounded by a stroma.

Traditionally established by neurobiologists to study neuronal electrical activities in brain slices, organotypic models have been implemented to monitor the dynamics of immune cells, first in lymphoid organs and then in tumors.^56^, ^121^, ^122^ Although the structure of the tumor is mostly preserved, one drawback is the difficulty to keep tumor explants several days in culture limiting the range of possibilities.

## 7 CONCLUDING REMARKS

CAR T cell therapy has revolutionized the treatment of patients with hematologic malignancies but not with solid tumors. Several different limitations to achieving a durable remission with CAR T cell therapy have recently been put forward. Here, we have reviewed the important advances recently made in the study of CAR T cell trafficking, highlighting the presence of obstacles that prevent engineered T cells from reaching cancer cells. We have also presented exciting approaches that are currently under development to increase the migration of CAR T cells into and within tumors with the overall objective of developing therapeutic cell products endowed with controlled potency and safety.

Despite considerable progress, many fundamental questions remain to be answered. For instance, what is the kinetic of tumor cells entry into tumor sites and tumor cell killing? What is the minimum amount of target antigens for CAR T cell recognition? Are CAR T cells able to kill multiple target cells? We believe that all these questions will be approached through the development of proper and relevant preclinical models and innovative imaging technologies.

Another area of future investigations concerns the use of CAR T cells besides tumors. Engineered T cells have also been recently applied in the fields of autoimmune diseases and fibrosis.^123^ How CAR T cells manage to migrate in these different environments is an open question. Whereas fibrotic sites might mimic tumors in terms of defective T cell migration, inflamed tissues might, in contrast, favor lymphocyte navigation.

We believe that all these issues and questions will be approached through the development of proper and relevant preclinical models, gene editing technologies, such as CRISPR‐Cas9 method,^124^ and innovative imaging technologies.

## AUTHORSHIP

All authors contributed to both the review conceptualization and the writing process.

## DISCLOSURES

The authors declare no conflicts of interest.

## References

[1] Golstein P , Griffiths GM . An early history of T cell‐mediated cytotoxicity. Nat Rev Immunol. 2018;18:527‐535. 29662120 10.1038/s41577-018-0009-3

[2] Gooden MJ , de Bock GH , Leffers N , Daemen T , Nijman HW . The prognostic influence of tumour‐infiltrating lymphocytes in cancer: a systematic review with meta‐analysis. Br J Cancer. 2011;105:93‐103. 21629244 10.1038/bjc.2011.189 PMC3137407

[3] Vinay DS , Ryan EP , Pawelec G , et al. Immune evasion in cancer: mechanistic basis and therapeutic strategies. Semin Cancer Biol. 2015;35(Suppl):S185‐S198. 25818339 10.1016/j.semcancer.2015.03.004

[4] Yang Y . Cancer immunotherapy: harnessing the immune system to battle cancer. J Clin Invest. 2015;125:3335‐3337. 26325031 10.1172/JCI83871 PMC4588312

[5] Herbst RS , Soria JC , Kowanetz M , et al. Predictive correlates of response to the anti‐PD‐L1 antibody MPDL3280A in cancer patients. Nature. 2014;515:563‐567. 25428504 10.1038/nature14011 PMC4836193

[6] van der Bruggen P , Traversari C , Chomez P , et al. A gene encoding an antigen recognized by cytolytic T lymphocytes on a human melanoma. Science. 1991;254:1643‐1647. 1840703 10.1126/science.1840703

[7] Gardner A , Ruffell B . Dendritic cells and cancer immunity. Trends Immunol. 2016;37:855‐865. 27793569 10.1016/j.it.2016.09.006 PMC5135568

[8] Hildner K , Edelson BT , Purtha WE , et al. Batf3 deficiency reveals a critical role for CD8alpha+ dendritic cells in cytotoxic T cell immunity. Science. 2008;322:1097‐1100. 19008445 10.1126/science.1164206 PMC2756611

[9] Ochsenbein AF , Sierro S , Odermatt B , et al. Roles of tumour localization, second signals and cross priming in cytotoxic T‐cell induction. Nature. 2001;411:1058‐1064. 11429607 10.1038/35082583

[10] Dieu‐Nosjean MC , Antoine M , Danel C , et al. Long‐term survival for patients with non‐small‐cell lung cancer with intratumoral lymphoid structures. J Clin Oncol. 2008;26:4410‐4417. 18802153 10.1200/JCO.2007.15.0284

[11] Schumacher TN , Scheper W , Kvistborg P . Cancer neoantigens. Annu Rev Immunol. 2019;37:173‐200. 30550719 10.1146/annurev-immunol-042617-053402

[12] Masopust D , Schenkel JM . The integration of T cell migration, differentiation and function. Nat Rev Immunol. 2013;13:309‐320. 23598650 10.1038/nri3442

[13] Slaney CY , Kershaw MH , Darcy PK . Trafficking of T cells into tumors. Cancer Res. 2014;74:7168‐7174. 25477332 10.1158/0008-5472.CAN-14-2458

[14] Headley MB , Bins A , Nip A , et al. Visualization of immediate immune responses to pioneer metastatic cells in the lung. Nature. 2016;531:513‐517. 26982733 10.1038/nature16985 PMC4892380

[15] Eyles J , Puaux AL , Wang X , et al. Tumor cells disseminate early, but immunosurveillance limits metastatic outgrowth, in a mouse model of melanoma. J Clin Invest. 2010;120:2030‐2039. 20501944 10.1172/JCI42002 PMC2877955

[16] Romero I , Garrido F , Garcia‐Lora AM . Metastases in immune‐mediated dormancy: a new opportunity for targeting cancer. Cancer Res. 2014;74:6750‐6757. 25411345 10.1158/0008-5472.CAN-14-2406

[17] Queirolo P , Spagnolo F , Ascierto PA , et al. Efficacy and safety of ipilimumab in patients with advanced melanoma and brain metastases. J Neurooncol. 2014;118:109‐116. 24532241 10.1007/s11060-014-1400-y PMC4023079

[18] Goldberg SB , Gettinger SN , Mahajan A , et al. Pembrolizumab for patients with melanoma or non‐small‐cell lung cancer and untreated brain metastases: early analysis of a non‐randomised, open‐label, phase 2 trial. Lancet Oncol. 2016;17:976‐983. 27267608 10.1016/S1470-2045(16)30053-5 PMC5526047

[19] Blank CU , Haining WN , Held W , et al. Defining ‘T cell exhaustion’. Nat Rev Immunol. 2019;19:665‐674. 31570879 10.1038/s41577-019-0221-9 PMC7286441

[20] McLane LM , Abdel‐Hakeem MS , Wherry EJ . CD8 T cell exhaustion during chronic viral infection and cancer. Annu Rev Immunol. 2019;37:457‐495. 30676822 10.1146/annurev-immunol-041015-055318

[21] Gattinoni L , Lugli E , Ji Y , et al. A human memory T cell subset with stem cell‐like properties. Nat Med. 2011;17:1290‐1297. 21926977 10.1038/nm.2446 PMC3192229

[22] Gattinoni L , Klebanoff CA , Restifo NP . Paths to stemness: building the ultimate antitumour T cell. Nat Rev Cancer. 2012;12:671‐684. 22996603 10.1038/nrc3322 PMC6352980

[23] Biasco L , Scala S , Basso Ricci L , et al. In vivo tracking of T cells in humans unveils decade‐long survival and activity of genetically modified T memory stem cells. Sci Transl Med. 2015;7:273ra13. 10.1126/scitranslmed.3010314 25653219

[24] Brummelman J , Mazza EMC , Alvisi G , et al. High‐dimensional single cell analysis identifies stem‐like cytotoxic CD8(+) T cells infiltrating human tumors. J Exp Med. 2018;215:2520‐2535. 30154266 10.1084/jem.20180684 PMC6170179

[25] Jansen CS , Prokhnevska N , Master VA , et al. An intra‐tumoral niche maintains and differentiates stem‐like CD8 T cells. Nature. 2019;576:465‐470. 31827286 10.1038/s41586-019-1836-5 PMC7108171

[26] Savas P , Virassamy B , Ye C , C. Kathleen Cuningham Foundation Consortium for Research into Familial Breast , et al. Single‐cell profiling of breast cancer T cells reveals a tissue‐resident memory subset associated with improved prognosis. Nat Med. 2018;24:986‐993. 29942092 10.1038/s41591-018-0078-7

[27] Rosenberg SA , Packard BS , Aebersold PM , et al. Use of tumor‐infiltrating lymphocytes and interleukin‐2 in the immunotherapy of patients with metastatic melanoma. A preliminary report. N Engl J Med. 1988;319:1676‐1680. 3264384 10.1056/NEJM198812223192527

[28] June CH , O'Connor RS , Kawalekar OU , Ghassemi S , Milone MC . CAR T cell immunotherapy for human cancer. Science. 2018;359:1361‐1365. 29567707 10.1126/science.aar6711

[29] Chang ZL , Chen YY . CARs: synthetic immunoreceptors for cancer therapy and beyond. Trends Mol Med. 2017;23:430‐450. 28416139 10.1016/j.molmed.2017.03.002 PMC5423782

[30] Guedan S , Ruella M , June CH . Emerging cellular therapies for cancer. Annu Rev Immunol. 2019;37:145‐171. 30526160 10.1146/annurev-immunol-042718-041407 PMC7399614

[31] Rafiq S , Hackett CS , Brentjens RJ . Engineering strategies to overcome the current roadblocks in CAR T cell therapy. Nat Rev Clin Oncol. 2020;17:147‐167. 31848460 10.1038/s41571-019-0297-y PMC7223338

[32] Lesch S , Benmebarek MR , Cadilha BL , et al. Determinants of response and resistance to CAR T cell therapy. Semin Cancer Biol. In press. https://doi.10.1016/j.semcancer.2019.11.004. 10.1016/j.semcancer.2019.11.004 31705998

[33] Gross G , Eshhar Z . Therapeutic potential of T cell chimeric antigen receptors (CARs) in cancer treatment: counteracting off‐tumor toxicities for safe CAR T cell therapy. Annu Rev Pharmacol Toxicol. 2016;56:59‐83. 26738472 10.1146/annurev-pharmtox-010814-124844

[34] Hartmann J , Schussler‐Lenz M , Bondanza A , Buchholz CJ . Clinical development of CAR T cells‐challenges and opportunities in translating innovative treatment concepts. EMBO Mol Med. 2017;9:1183‐1197. 28765140 10.15252/emmm.201607485 PMC5582407

[35] Fucà G , Reppel L , Landoni E , Savoldo B , Dotti G . Enhancing Chimeric Antigen Receptor T‐Cell Efficacy in Solid Tumors. Clin Cancer Res. 2020;26:2444‐2451. 32015021 10.1158/1078-0432.CCR-19-1835 PMC7269829

[36] Ahmed N , Brawley VS , Hegde M , et al. Human epidermal growth factor receptor 2 (HER2) ‐specific chimeric antigen receptor‐modified T cells for the immunotherapy of HER2‐positive sarcoma. J Clin Oncol. 2015;33:1688‐1696. 25800760 10.1200/JCO.2014.58.0225 PMC4429176

[37] Beatty GL , O'Hara MH , Lacey SF , et al. Activity of mesothelin‐specific chimeric antigen receptor T cells against pancreatic carcinoma metastases in a phase 1 trial. Gastroenterology. 2018;155:29‐32. 29567081 10.1053/j.gastro.2018.03.029 PMC6035088

[38] Morgan RA , Yang JC , Kitano M , Dudley ME , Laurencot CM , Rosenberg SA . Case report of a serious adverse event following the administration of T cells transduced with a chimeric antigen receptor recognizing ERBB2. Mol Ther. 2010;18:843‐851. 20179677 10.1038/mt.2010.24 PMC2862534

[39] Bernhard H , Neudorfer J , Gebhard K , et al. Adoptive transfer of autologous, HER2‐specific, cytotoxic T lymphocytes for the treatment of HER2‐overexpressing breast cancer. Cancer Immunol Immunother. 2008;57:271‐280. 17646988 10.1007/s00262-007-0355-7 PMC11030865

[40] Peranzoni E , Lemoine J , Vimeux L , et al. Macrophages impede CD8 T cells from reaching tumor cells and limit the efficacy of anti‐PD‐1 treatment. Proc Natl Acad Sci U S A. 2018;115:E4041‐E4050. 29632196 10.1073/pnas.1720948115 PMC5924916

[41] Dustin ML . Integrins and their role in immune cell adhesion. Cell. 2019;177:499‐501. 30952447 10.1016/j.cell.2019.03.038

[42] Franciszkiewicz K , Le Floc'h A , Boutet M , Vergnon I , Schmitt A , Mami‐Chouaib F . CD103 or LFA‐1 engagement at the immune synapse between cytotoxic T cells and tumor cells promotes maturation and regulates T‐cell effector functions. Cancer Res. 2013;73:617‐628. 23188505 10.1158/0008-5472.CAN-12-2569

[43] Liadi I , Singh H , Romain G , et al. Individual motile CD4(+) T cells can participate in efficient multikilling through conjugation to multiple tumor cells. Cancer Immunol Res. 2015;3:473‐482. 25711538 10.1158/2326-6066.CIR-14-0195 PMC4421910

[44] Davenport AJ , Cross RS , Watson KA , et al. Chimeric antigen receptor T cells form nonclassical and potent immune synapses driving rapid cytotoxicity. Proc Natl Acad Sci U S A. 2018;115:E2068‐E2076. 29440406 10.1073/pnas.1716266115 PMC5834689

[45] Cazaux M , Grandjean CL , Lemaitre F , et al. Single‐cell imaging of CAR T cell activity in vivo reveals extensive functional and anatomical heterogeneity. J Exp Med. 2019;216:1038‐1049. 30936262 10.1084/jem.20182375 PMC6504219

[46] Fraietta JA , Nobles CL , Sammons MA , et al. Disruption of TET2 promotes the therapeutic efficacy of CD19‐targeted T cells. Nature. 2018;558:307‐312. 29849141 10.1038/s41586-018-0178-z PMC6320248

[47] Lynn RC , Weber EW , Sotillo E , et al. c‐Jun overexpression in CAR T cells induces exhaustion resistance. Nature. 2019;576:293‐300. 31802004 10.1038/s41586-019-1805-z PMC6944329

[48] Crompton JG , Sukumar M , Restifo NP . Uncoupling T‐cell expansion from effector differentiation in cell‐based immunotherapy. Immunol Rev. 2014;257:264‐276. 24329803 10.1111/imr.12135 PMC3915736

[49] Klein D . The tumor vascular endothelium as decision maker in cancer therapy. Front Oncol. 2018;8:367. 30250827 10.3389/fonc.2018.00367 PMC6139307

[50] Bergers G , Song S . The role of pericytes in blood‐vessel formation and maintenance. Neuro Oncol. 2005;7:452‐464. 16212810 10.1215/S1152851705000232 PMC1871727

[51] Piali L , Fichtel A , Terpe HJ , Imhof BA , Gisler RH . Endothelial vascular cell adhesion molecule 1 expression is suppressed by melanoma and carcinoma. J Exp Med. 1995;181:811‐816. 7530765 10.1084/jem.181.2.811 PMC2191895

[52] Griffioen AW , Damen CA , Blijham GH , Groenewegen G . Tumor angiogenesis is accompanied by a decreased inflammatory response of tumor‐associated endothelium. Blood. 1996;88:667‐673. 8695814

[53] Sellmyer MA , Richman SA , Lohith K , et al. Imaging CAR T cell trafficking with eDHFR as a PET reporter gene. Mol Ther. 2020;28:42‐51. 31668558 10.1016/j.ymthe.2019.10.007 PMC6953896

[54] Joyce JA , Fearon DT . T cell exclusion, immune privilege, and the tumor microenvironment. Science. 2015;348:74‐80. 25838376 10.1126/science.aaa6204

[55] Yamauchi M , Barker TH , Gibbons DL , Kurie JM . The fibrotic tumor stroma. J Clin Invest. 2018;128:16‐25. 29293090 10.1172/JCI93554 PMC5749516

[56] Salmon H , Franciszkiewicz K , Damotte D , et al. Matrix architecture defines the preferential localization and migration of T cells into the stroma of human lung tumors. J Clin Invest. 2012;122:899‐910. 22293174 10.1172/JCI45817 PMC3287213

[57] Peranzoni E , Zilio S , Marigo I , et al. Myeloid‐derived suppressor cell heterogeneity and subset definition. Curr Opin Immunol. 2010;22:238‐244. 20171075 10.1016/j.coi.2010.01.021

[58] Engelhardt JJ , Boldajipour B , Beemiller P , et al. Marginating dendritic cells of the tumor microenvironment cross‐present tumor antigens and stably engage tumor‐specific T cells. Cancer Cell. 2012;21:402‐417. 22439936 10.1016/j.ccr.2012.01.008 PMC3311997

[59] Sugiura A , Rathmell JC . Metabolic barriers to T cell function in tumors. J Immunol. 2018;200:400‐407. 29311381 10.4049/jimmunol.1701041 PMC5777533

[60] Manaster Y , Shipony Z , Hutzler A , et al. Reduced CTL motility and activity in avascular tumor areas. Cancer Immunol Immunother. 2019;68:1287‐1301. 31253998 10.1007/s00262-019-02361-5 PMC11028152

[61] Jayaprakash P , Ai M , Liu A , et al. Targeted hypoxia reduction restores T cell infiltration and sensitizes prostate cancer to immunotherapy. J Clin Invest. 2018;128:5137‐5149. 30188869 10.1172/JCI96268 PMC6205399

[62] Kawalekar OU , RS OC , Fraietta JA , et al. Distinct signaling of coreceptors regulates specific metabolism pathways and impacts memory development in CAR T cells. Immunity. 2016;44:712. 10.1016/j.immuni.2016.02.023 28843072

[63] Mollica Poeta V , Massara M , Capucetti A , Bonecchi R . Chemokines and chemokine receptors: new targets for cancer immunotherapy. Front Immunol. 2019;10:379. 30894861 10.3389/fimmu.2019.00379 PMC6414456

[64] Dangaj D , Bruand M , Grimm AJ , et al. Cooperation between constitutive and inducible chemokines enables T cell engraftment and immune attack in solid tumors. Cancer Cell. 2019;35:885‐900e10. 31185212 10.1016/j.ccell.2019.05.004 PMC6961655

[65] Spranger S , Bao R , Gajewski TF . Melanoma‐intrinsic beta‐catenin signalling prevents anti‐tumour immunity. Nature. 2015;523:231‐235. 25970248 10.1038/nature14404

[66] Feig C , Jones JO , Kraman M , et al. Targeting CXCL12 from FAP‐expressing carcinoma‐associated fibroblasts synergizes with anti‐PD‐L1 immunotherapy in pancreatic cancer. Proc Natl Acad Sci U S A. 2013;110:20212‐20217. 24277834 10.1073/pnas.1320318110 PMC3864274

[67] Shah NN , Fry TJ . Mechanisms of resistance to CAR T cell therapy. Nat Rev Clin Oncol. 2019;16:372‐385. 30837712 10.1038/s41571-019-0184-6 PMC8214555

[68] Fry TJ , Shah NN , Orentas RJ , et al. CD22‐targeted CAR T cells induce remission in B‐ALL that is naive or resistant to CD19‐targeted CAR immunotherapy. Nat Med. 2018;24:20‐28. 29155426 10.1038/nm.4441 PMC5774642

[69] Hamieh M , Dobrin A , Cabriolu A , et al. CAR T cell trogocytosis and cooperative killing regulate tumour antigen escape. Nature. 2019;568:112‐116. 30918399 10.1038/s41586-019-1054-1 PMC6707377

[70] Ramakrishna S , Highfill SL , Walsh Z , et al. Modulation of target antigen density improves CAR T‐cell functionality and persistence. Clin Cancer Res. 2019;25:5329‐5341. 31110075 10.1158/1078-0432.CCR-18-3784 PMC8290499

[71] Huppa JB , Axmann M , Mortelmaier MA , et al. TCR‐peptide‐MHC interactions in situ show accelerated kinetics and increased affinity. Nature. 2010;463:963‐967. 20164930 10.1038/nature08746 PMC3273423

[72] Majzner RG , Rietberg SP , Sotillo E , et al. Tuning the antigen density requirement for CAR T‐cell activity. Cancer Discov. 2020;10:702‐723. 32193224 10.1158/2159-8290.CD-19-0945 PMC7939454

[73] Pont MJ , Hill T , Cole GO , et al. gamma‐Secretase inhibition increases efficacy of BCMA‐specific chimeric antigen receptor T cells in multiple myeloma. Blood. 2019;134:1585‐1597. 31558469 10.1182/blood.2019000050 PMC6871311

[74] Neelapu SS , Tummala S , Kebriaei P , et al. Chimeric antigen receptor T‐cell therapy ‐ assessment and management of toxicities. Nat Rev Clin Oncol. 2018;15:47‐62. 28925994 10.1038/nrclinonc.2017.148 PMC6733403

[75] Taraseviciute A , Tkachev V , Ponce R , et al. Chimeric antigen receptor T cell‐mediated neurotoxicity in nonhuman primates. Cancer Discov. 2018;8:750‐763. 29563103 10.1158/2159-8290.CD-17-1368 PMC6058704

[76] Gust J , Hay KA , Hanafi LA , et al. Endothelial activation and blood‐brain barrier disruption in neurotoxicity after adoptive immunotherapy with CD19 CAR‐T cells. Cancer Discov. 2017;7:1404‐1419. 29025771 10.1158/2159-8290.CD-17-0698 PMC5718945

[77] Lee DW , Kochenderfer JN , Stetler‐Stevenson M , et al. T cells expressing CD19 chimeric antigen receptors for acute lymphoblastic leukaemia in children and young adults: a phase 1 dose‐escalation trial. Lancet. 2015;385:517‐528. 25319501 10.1016/S0140-6736(14)61403-3 PMC7065359

[78] Casucci M , Hawkins RE , Dotti G , Bondanza A . Overcoming the toxicity hurdles of genetically targeted T cells. Cancer Immunol Immunother. 2015;64:123‐130. 25488419 10.1007/s00262-014-1641-9 PMC11028535

[79] Hamzah J , Jugold M , Kiessling F , et al. Vascular normalization in Rgs5‐deficient tumours promotes immune destruction. Nature. 2008;453:410‐414. 18418378 10.1038/nature06868

[80] Shrimali RK , Yu Z , Theoret MR , Chinnasamy D , Restifo NP , Rosenberg SA . Antiangiogenic agents can increase lymphocyte infiltration into tumor and enhance the effectiveness of adoptive immunotherapy of cancer. Cancer Res. 2010;70:6171‐6180. 20631075 10.1158/0008-5472.CAN-10-0153 PMC2912959

[81] Schmittnaegel M , Rigamonti N , Kadioglu E , et al. Dual angiopoietin‐2 and VEGFA inhibition elicits antitumor immunity that is enhanced by PD‐1 checkpoint blockade. Sci Transl Med. 2017;9:eaak9670. 28404865 10.1126/scitranslmed.aak9670

[82] de Aguiar RB , de Moraes JZ . Exploring the immunological mechanisms underlying the anti‐vascular endothelial growth factor activity in tumors. Front Immunol. 2019;10:1023. 31156623 10.3389/fimmu.2019.01023 PMC6530399

[83] Guipaud O , Jaillet C , Clement‐Colmou K , Francois A , Supiot S , Milliat F . The importance of the vascular endothelial barrier in the immune‐inflammatory response induced by radiotherapy. Br J Radiol. 2018;91:20170762. 29630386 10.1259/bjr.20170762 PMC6223160

[84] Lindau D , Gielen P , Kroesen M , Wesseling P , Adema GJ . The immunosuppressive tumour network: myeloid‐derived suppressor cells, regulatory T cells and natural killer T cells. Immunology. 2013;138:105‐115. 23216602 10.1111/imm.12036 PMC3575763

[85] Simpson TR , Li F , Montalvo‐Ortiz W , et al. Fc‐dependent depletion of tumor‐infiltrating regulatory T cells co‐defines the efficacy of anti‐CTLA‐4 therapy against melanoma. J Exp Med. 2013;210:1695‐1710. 23897981 10.1084/jem.20130579 PMC3754863

[86] Li X , Kostareli E , Suffner J , Garbi N , Hammerling GJ . Efficient Treg depletion induces T‐cell infiltration and rejection of large tumors. Eur J Immunol. 2010;40:3325‐3335. 21072887 10.1002/eji.201041093

[87] Beatty GL , Winograd R , Evans RA , et al. Exclusion of T cells from pancreatic carcinomas in mice is regulated by Ly6C(low) F4/80(+) extratumoral macrophages. Gastroenterology. 2015;149:201‐210. 25888329 10.1053/j.gastro.2015.04.010 PMC4478138

[88] Giavridis T , van der Stegen SJC , Eyquem J , Hamieh M , Piersigilli A , Sadelain M . CAR T cell‐induced cytokine release syndrome is mediated by macrophages and abated by IL‐1 blockade. Nat Med. 2018;24:731‐738. 29808005 10.1038/s41591-018-0041-7 PMC6410714

[89] Bercovici N , Guerin MV , Trautmann A , Donnadieu E . The remarkable plasticity of macrophages: a chance to fight cancer. Front Immunol. 2019;10:1563. 31354719 10.3389/fimmu.2019.01563 PMC6640155

[90] Kakarla S , Chow KK , Mata M , et al. Antitumor effects of chimeric receptor engineered human T cells directed to tumor stroma. Mol Ther. 2013;21:1611‐1620. 23732988 10.1038/mt.2013.110 PMC3734659

[91] Caruana I , Savoldo B , Hoyos V , et al. Heparanase promotes tumor infiltration and antitumor activity of CAR‐redirected T lymphocytes. Nat Med. 2015;21:524‐529. 25849134 10.1038/nm.3833 PMC4425589

[92] Lee Y , Auh SL , Wang Y , et al. Therapeutic effects of ablative radiation on local tumor require CD8+ T cells: changing strategies for cancer treatment. Blood. 2009;114:589‐595. 19349616 10.1182/blood-2009-02-206870 PMC2713472

[93] Seyedin SN , Schoenhals JE , Lee DA , et al. Strategies for combining immunotherapy with radiation for anticancer therapy. Immunotherapy. 2015;7:967‐980. 26310908 10.2217/imt.15.65 PMC4825325

[94] Xu J , Wang Y , Shi J , Liu J , Li Q , Chen L . Combination therapy: a feasibility strategy for CAR‐T cell therapy in the treatment of solid tumors. Oncol Lett. 2018;16:2063‐2070. 30008901 10.3892/ol.2018.8946 PMC6036511

[95] Hassan R , Williams‐Gould J , Steinberg SM , et al. Tumor‐directed radiation and the immunotoxin SS1P in the treatment of mesothelin‐expressing tumor xenografts. Clin Cancer Res. 2006;12:4983‐4988. 16914588 10.1158/1078-0432.CCR-06-0441

[96] Cao N , Li S , Wang Z , et al. NF‐kappaB‐mediated HER2 overexpression in radiation‐adaptive resistance. Radiat Res. 2009;171:9‐21. 19138055 10.1667/RR1472.1 PMC2659759

[97] Mule JJ , Custer M , Averbook B , et al. RANTES secretion by gene‐modified tumor cells results in loss of tumorigenicity in vivo: role of immune cell subpopulations. Hum Gene Ther. 1996;7:1545‐1553. 8864755 10.1089/hum.1996.7.13-1545

[98] Kershaw MH , Wang G , Westwood JA , et al. Redirecting migration of T cells to chemokine secreted from tumors by genetic modification with CXCR2. Hum Gene Ther. 2002;13:1971‐1980. 12427307 10.1089/10430340260355374

[99] Craddock JA , Lu A , Bear A , et al. Enhanced tumor trafficking of GD2 chimeric antigen receptor T cells by expression of the chemokine receptor CCR2b. J Immunother. 2010;33:780‐788. 20842059 10.1097/CJI.0b013e3181ee6675 PMC2998197

[100] Moon EK , Carpenito C , Sun J , et al. Expression of a functional CCR2 receptor enhances tumor localization and tumor eradication by retargeted human T cells expressing a mesothelin‐specific chimeric antibody receptor. Clin Cancer Res. 2011;17:4719‐4730. 21610146 10.1158/1078-0432.CCR-11-0351 PMC3612507

[101] Whilding LM , Halim L , Draper B , et al. CAR T‐cells targeting the integrin αvβ6 and co‐expressing the chemokine receptor CXCR2 demonstrate enhanced homing and efficacy against several solid malignancies. Cancers (Basel). 2019;11:674‐690. 10.3390/cancers11050674 PMC6563120 31091832

[102] Di Stasi A , De Angelis B , Rooney CM , et al. T lymphocytes coexpressing CCR4 and a chimeric antigen receptor targeting CD30 have improved homing and antitumor activity in a Hodgkin tumor model. Blood. 2009;113:6392‐6402. 19377047 10.1182/blood-2009-03-209650 PMC2710932

[103] Moon EK , Wang LS , Bekdache K , et al. Intra‐tumoral delivery of CXCL11 via a vaccinia virus, but not by modified T cells, enhances the efficacy of adoptive T cell therapy and vaccines. Oncoimmunology. 2018;7:e1395997. 29399394 10.1080/2162402X.2017.1395997 PMC5790399

[104] Newick K , O'Brien S , Sun J , et al. Augmentation of CAR T‐cell trafficking and antitumor efficacy by blocking protein kinase a localization. Cancer Immunol Res. 2016;4:541‐551. 27045023 10.1158/2326-6066.CIR-15-0263 PMC4891259

[105] Schmidts A , Maus MV . Making CAR T cells a solid option for solid tumors. Front Immunol. 2018;9:2593. 30467505 10.3389/fimmu.2018.02593 PMC6235951

[106] Springuel L , Lonez C , Alexandre B , et al. Chimeric antigen receptor‐T cells for targeting solid tumors: current challenges and existing strategies. BioDrugs. 2019;33:515‐537. 31363930 10.1007/s40259-019-00368-z PMC6790340

[107] Brown CE , Aguilar B , Starr R , et al. Optimization of IL13Ralpha2‐targeted chimeric antigen receptor T cells for improved anti‐tumor efficacy against glioblastoma. Mol Ther. 2018;26:31‐44. 29103912 10.1016/j.ymthe.2017.10.002 PMC5763077

[108] Brown CE , Alizadeh D , Starr R , et al. Regression of glioblastoma after chimeric antigen receptor T‐cell therapy. N Engl J Med. 2016;375:2561‐2569. 28029927 10.1056/NEJMoa1610497 PMC5390684

[109] Donovan LK , Delaidelli A , Joseph SK , et al. Locoregional delivery of CAR T cells to the cerebrospinal fluid for treatment of metastatic medulloblastoma and ependymoma. Nat Med. 2020;26:720‐731. 32341580 10.1038/s41591-020-0827-2 PMC8815773

[110] Theruvath J , Sotillo E , Mount CW , et al. Locoregionally administered B7‐H3‐targeted CAR T cells for treatment of atypical teratoid/rhabdoid tumors. Nat Med. 2020;26:712‐719. 32341579 10.1038/s41591-020-0821-8 PMC7992505

[111] Hegde PS , Chen DS . Top 10 challenges in cancer immunotherapy. Immunity. 2020;52:17‐35. 31940268 10.1016/j.immuni.2019.12.011

[112] Davenport AJ , Jenkins MR , Cross RS , et al. CAR‐T cells inflict sequential killing of multiple tumor target cells. Cancer Immunol Res. 2015;3:483‐494. 25711536 10.1158/2326-6066.CIR-15-0048

[113] Emami‐Shahri N , Foster J , Kashani R , et al. Clinically compliant spatial and temporal imaging of chimeric antigen receptor T‐cells. Nat Commun. 2018;9:1081. 29540684 10.1038/s41467-018-03524-1 PMC5852048

[114] Norelli M , Camisa B , Barbiera G , et al. Monocyte‐derived IL‐1 and IL‐6 are differentially required for cytokine‐release syndrome and neurotoxicity due to CAR T cells. Nat Med. 2018;24:739‐748. 29808007 10.1038/s41591-018-0036-4

[115] Mulazzani M , Frassle SP , von Mucke‐Heim I , et al. Long‐term in vivo microscopy of CAR T cell dynamics during eradication of CNS lymphoma in mice. Proc Natl Acad Sci U S A. 2019;116:24275‐24284. 31712432 10.1073/pnas.1903854116 PMC6883823

[116] Siegler EL , Wang P . Preclinical models in chimeric antigen receptor‐engineered T‐cell therapy. Hum Gene Ther. 2018;29:534‐546. 29390873 10.1089/hum.2017.243

[117] Pascoal S , Salzer B , Scheuringer E , et al. A preclinical embryonic zebrafish xenograft model to investigate CAR T cells in vivo. Cancers (Basel). 2020;12:567‐580. 10.3390/cancers12030567 PMC7139560 32121414

[118] Schnalzger TE , de Groot MH , Zhang C , et al. 3D model for CAR‐mediated cytotoxicity using patient‐derived colorectal cancer organoids. EMBO J. 2019;38:e100928. 31036555 10.15252/embj.2018100928 PMC6576164

[119] Wallstabe L , Gottlich C , Nelke LC , et al. ROR1‐CAR T cells are effective against lung and breast cancer in advanced microphysiologic 3D tumor models. JCI Insight. 2019;4:e126345. 10.1172/jci.insight.126345 PMC6795380 31415244

[120] Jacob F , Salinas RD , Zhang DY , et al. A patient‐derived glioblastoma organoid model and biobank recapitulates inter‐ and intra‐tumoral heterogeneity. Cell. 2020;180:188‐204e22. 31883794 10.1016/j.cell.2019.11.036 PMC7556703

[121] Bhakta NR , Oh DY , Lewis RS . Calcium oscillations regulate thymocyte motility during positive selection in the three‐dimensional thymic environment. Nat Immunol. 2005;6:143‐151. 15654342 10.1038/ni1161

[122] Asperti‐Boursin F , Real E , Bismuth G , Trautmann A , Donnadieu E . CCR7 ligands control basal T cell motility within lymph node slices in a phosphoinositide 3‐kinase‐independent manner. J Exp Med. 2007;204:1167‐1179. 17485513 10.1084/jem.20062079 PMC2118589

[123] Weber EW , Maus MV , Mackall CL . The emerging landscape of immune cell therapies. Cell. 2020;181:46‐62. 32243795 10.1016/j.cell.2020.03.001 PMC8900215

[124] Stadtmauer EA , Fraietta JA , Davis MM , et al. CRISPR‐engineered T cells in patients with refractory cancer. Science. 2020;367:eaba7365. 32029687 10.1126/science.aba7365 PMC11249135

